# Antifungal Effect of Polygodial on *Botrytis cinerea*, a Fungal Pathogen Affecting Table Grapes

**DOI:** 10.3390/ijms18112251

**Published:** 2017-10-27

**Authors:** Héctor Carrasco, Christian Robles-Kelly, Julia Rubio, Andrés F. Olea, Rolando Martínez, Evelyn Silva-Moreno

**Affiliations:** 1 Instituto de Ciencias Químicas Aplicadas, Facultad de Ingeniería, Universidad Autónoma de Chile, El Llano Subercaseaux 2801, San Miguel, Santiago 8900000, Chile; hector.carrasco@uautonoma.cl (H.C.); andres.olea@uautonoma.cl (A.F.O.); 2 Instituto de Ciencias Biomédicas, Facultad de Medicina, Universidad Autónoma de Chile, El Llano Subercaseaux 2801, San Miguel, Santiago 8900000, Chile; christian.roblesk@gmail.com (C.R.-K.); jrubioa@gmail.com (J.R.); 3 Departamento de Ciencias Químicas, Facultad de Ciencias Exactas, Universidad Andrés Bello, Quillota 910, Viña del Mar 2520000, Chile; rmartinez@unab.cl

**Keywords:** *Botrytis cinerea*, polygodial, antifungal activity

## Abstract

The antifungal activity of polygodial, a secondary metabolite extracted from Canelo, on mycelial growth of different *Botrytis cinerea* isolates has been evaluated. The results show that polygodial affects growth of normal and resistant isolates of *B. cinerea* with EC_50_ values ranging between 117 and 175 ppm. In addition, polygodial markedly decreases the germination of *B. cinerea*, i.e., after six hours of incubation the percentage of germination decreases from 92% (control) to 25% and 5% in the presence of 20 ppm and 80 ppm of polygodial, respectively. Morphological studies indicate that conidia treated with polygodial are smaller, with irregular membrane border, and a lot of cell debris, as compared to conidia in the control. The existence of polygodial-induced membrane damage was confirmed by SYTOX^®^ Green uptake assay. Gene expression studies confirm that the effect of polygodial on *B. cinerea* is mainly attributed to inhibition of germination and appears at early stages of *B. cinerea* development. On the other hand, drimenol, a drimane with chemical structure quite similar to polygodial, inhibits the mycelial growth efficiently. Thus, both compounds inhibit mycelial growth by different mechanisms. The different antifungal activities of these compounds are discussed in terms of the electronic density on the double bond.

## 1. Introduction

*Botrytis cinerea*, also known as “gray mold fungus”, causes serious pre- and post-harvest diseases in at least 240 plant species [1], including agronomically-important crops. The broad host range of *B. cinerea* results in great economic losses not only during growth, but also during storage and transport of Chilean table grape exports. Thus, *B. cinerea* is a serious problem for the Chilean fruit industry because very large losses are associated with diseases produced by this pathogen.

Traditionally, control of *B. cinerea* is based on the application of chemical botrycides in order to prevent infection and to minimize postharvest losses. The main synthetic fungicides present a variety of modes of action. However, the magnitude and frequency of fungicidal treatments has induced the appearance of resistant isolates of this fungus [2]. In Chile, resistant isolates have been reported [3,4,5]. As a consequence, in many countries and as well in Chile, the regulatory authorities have started to restrict the use of chemical pesticides. Due to the enormous economical relevance of *B. cinerea* and the already mentioned problems in disease management, it is very important to find new compounds with activity against this pathogen. Development of resistance to chemical fungicides has prompted the search of new and efficient alternatives to control this fungus.

A common approach to explore and to find new molecules exhibiting biological activity is to study secondary metabolites produced by plants. Natural antifungal molecules may affect directly growth or germination of fungus, or as plant activators inducing plant resistance. A potential synergism effect of both activities could contribute to a reduction of fungicides input in crop protection [6].

**Figure 1:**
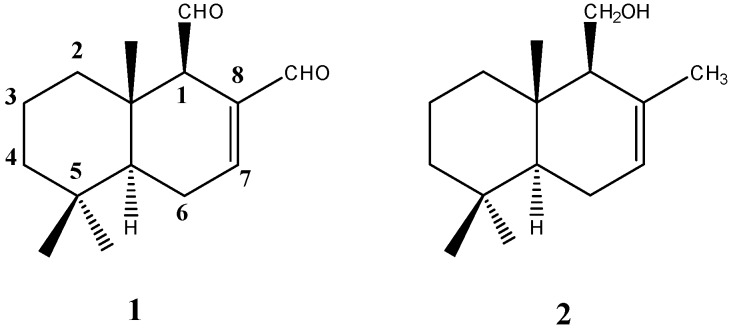
Chemical structures of polygodial (**1**) and drimenol (**2**).

Polygodial, a drimane sesquiterpene dialdehyde (Figure 1), originally isolated from the plant *Polygonum hydropiper* [7] and subsequently from *Warburgia ugandensis*, *Warburgia stuhlmannii*, *Pseudo-wintera colorata*, and *Drymis winteri* exhibits insect antifeedant activity [8], antimicrobial activity [9,10], and antinociceptive effect [11]. The antimicrobial activity of polygodial has been reported in bacteria, and fungi and yeast [12,13,14]. The fungicidal mechanism of polygodial was investigated in *Saccharomyces cerevisiae*, and it has been reported that plasma membrane disruption is the mechanism of polygodial-induced cell death [15]. Unlike many other antifungal agents, polygodial has fungicidal activity against yeasts and filamentous fungi [16]. Previous studies indicate that the presence of α,β-unsaturated aldehyde moiety is required for antifungal activity [17]. However, a recent study carried out on a group of sesquiterpenes and derivatives indicates that the presence of the Δ^7,8^-double bond is necessary for the activity against yeast, even though the activity is much higher when this double bond is forming part of the C-8 α,β-unsaturated aldehyde moiety [18]. Recently, we have shown that the presence of the ∆^7,8^-double bond in drimenol (Figure 1) and its derivatives is critical for antifungal activity against *B. cinerea* [19].

## 2. Results

### 2.1. Evaluation of Antifungal Activity of Polygodial on Botrytis cinerea

**Figure 2:**
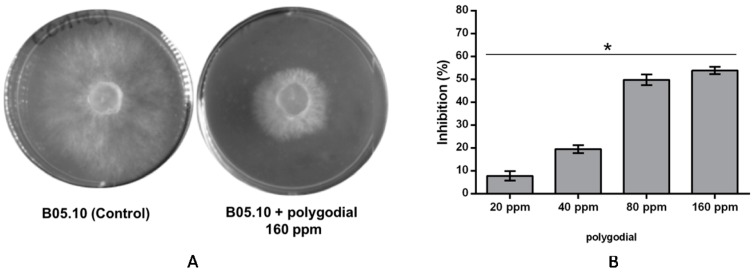
Effect of polygodial on mycelial growth of *Botrytis cinerea* in solid media. (**A**) Photographs of hyphal growth of *B. cinerea* in the absence and presence of 160 ppm of polygodial, respectively; and (**B**) the percentage of growth inhibition determined after 48 h of incubation at different concentrations of polygodial, i.e., 20, 40, 80 and 160 ppm. Each bar represents the average of at least three independent experiments ± standard deviation. * indicates significant differences between each polygodial concentration, evaluated by Tukey’s test; *p* < 0.05.

In order to evaluate the fungicide effect of polygodial on different isolates of *B. cinerea*, the inhibition of mycelial growth by different concentrations of polygodial was measured. The antifungal activity of polygodial was evaluated in radial growth measurements in malt-yeast media (Figure 2).

The results indicate that polygodial is able to affect *Botrytis* growth. However, examination of mycelia shows that no morphological changes in the mycelium or hyphae are produced by this compound (Figure 2A). In Figure 2B, the percentage of inhibition of hyphal growth is shown at different polygodial concentrations. The results indicate that polygodial reduces the *B. cinerea* mycelium growth by nearly 50% after two days of incubation at 80 and 160 ppm, and an EC_50_ value of 117 ppm has been obtained, which is higher than those reported for drimenol and its derivatives (80 ppm) and for a commercial natural fungicide, BC-1000 (30 ppm) [19].

### 2.2. Effect of Polygodial on Growth of Isolates of B. cinerea with Different Degrees of Sensitivity to Fungicides Commercially Used

**Figure 3:**
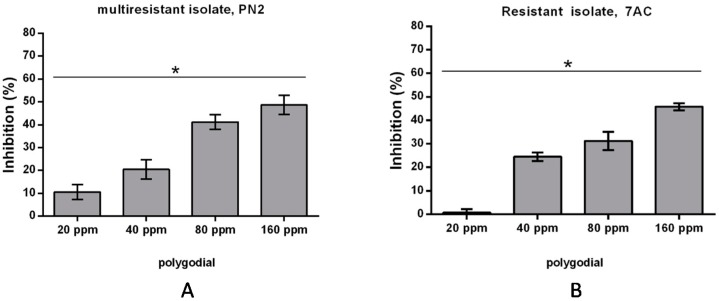
Effect of polygodial on mycelial growth of native *B. cinerea* with different degrees of sensitivity to commercial fungicides. Percentage of growth inhibition determined after 48 h of incubation at different concentrations of polygodial, i.e., 20, 40, 80 and 160 ppm against: (**A**) multiresistant (PN2); and (**B**) resistant (7AC) *B. cinerea* isolates. Each bar represents the average of at least three independent experiments ± standard deviation. (*) denotes significant differences between all polygodial concentrations evaluated by Tukey’s test; *p* < 0.05.

With the purpose to extend this study to resistant strains of *B. cinerea*, various native isolates presenting different degrees of sensitivity to commercial fungicides, were also used. Resistant isolates are PN2 (multiresistant strain) and 7AC (resistant strain for fenhexamid), obtained from cherry and pear, respectively. The characteristics of each isolate are described in Table 1 (Material and Methods Section). The results of mycelial growth inhibition are shown in Figure 3.

The EC_50_ values obtained for PN2 and 7AC are 168 and 175 ppm, respectively, which are similar to the EC_50_ value measured for B05.10.

### 2.3. Effect of Polygodial on Germination of B. cinerea

**Figure 4:**
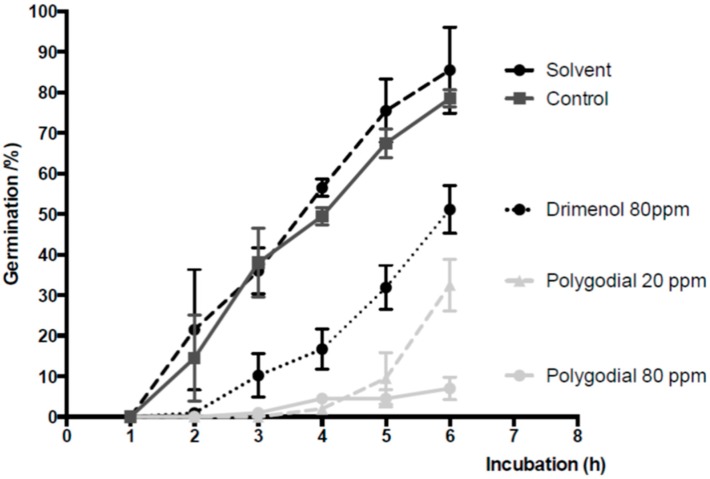
Effect of polygodial on conidial germination of *B. cinerea*. Plots represent percentage of germination of *B. cinerea* as a function of time in presence of polygodial, 20 ppm (▲) and 80 ppm (●); and drimenol 80 ppm (-●-) between 1 and 6 h post incubation. Final solvent concentration was identical in control and treatment assays. Each point represents the means of at least three independent experiments ± standard deviation. Drimenol data are from Robles-Kelly et al. [19].

The effect of polygodial on germination of *B. cinerea* has also been assessed and the results are shown in Figure 4. Germination studies were performed in presence of different concentrations of this compound and the percent of germination was quantified. The data indicate that germination of *B. cinerea* is strongly reduced in presence of polygodial and this effect increases with exposition time and polygodial concentration. After six hours of incubation, the percentage of germination decreases from 92% (control) to 25% and 5% at 20 ppm and 80 ppm of polygodial concentration, respectively.

**Figure 5:**
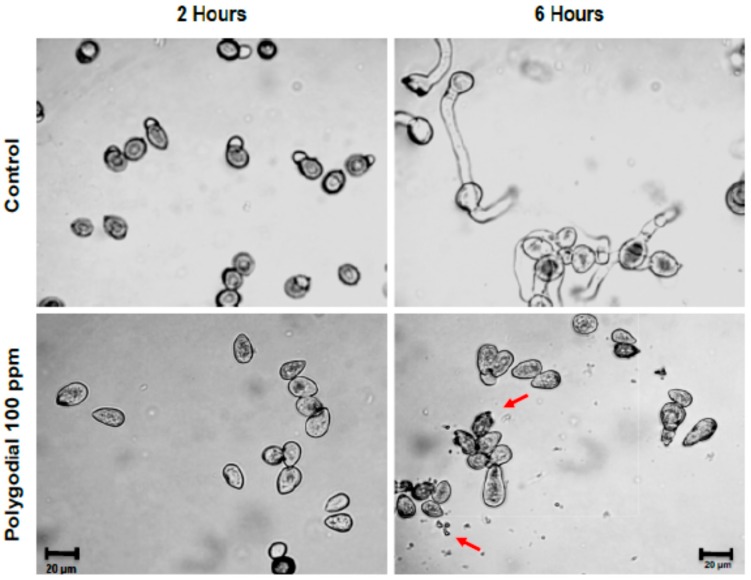
Morphological effect of polygodial on conidial germination of *B. cinerea*. Microphotographs correspond to *B. cinerea* germination in presence of 100 ppm of polygodial at 2 and 6 h post incubation. Final solvent concentration was identical in the control and treatment assays. Each bar corresponds to 20 µm. The red arrows indicate cell debris.

In addition, morphological studies of germination show that polygodial produces morphological changes in the conidia. In Figure 5 it is seen that after 6 h of incubation conidia treated with polygodial (100 ppm) are smaller than conidia in the control, with irregular membrane border, and a large amount of cell debris (red arrows, Figure 5). All these results suggest the existence of membrane damage induced by polygodial.

### 2.4. Mode of Action of Polygodial on Botrytis cinerea

In order to elucidate the mechanism by which polygodial is affecting *B. cinerea*, the membrane integrity, reactive oxygen species production, and gene expression of specific genes were studied at early stages of germination.

**Figure 6:**
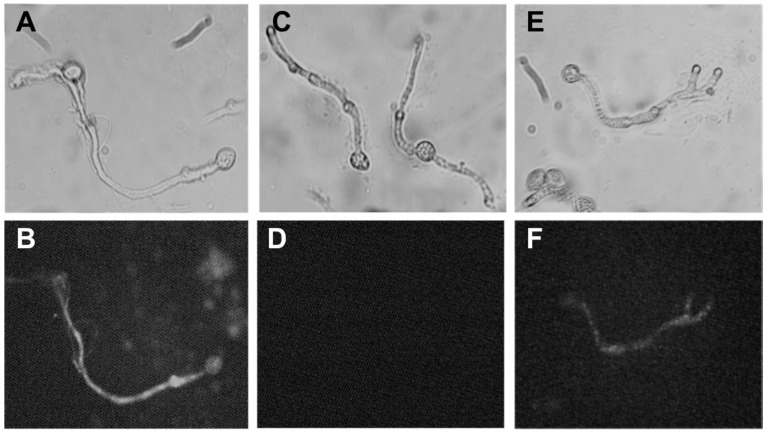
Effect of polygodial on membrane integrity of *B. cinerea*. Conidia (1 × 10^5^ conidia/mL) were incubated in liquid minimum medium in the presence of (**A**,**B**) 70% (*v*/*v*) ethanol, positive control; (**C**,**D**) methanol, negative control; (**E**,**F**) polygodial at 117 ppm, respectively for 1 h. The fluorescence of *B. cinerea* hyphae stained with SYTOX Green was observed using a fluorescence microscope. These photographs are representatives of five independent experiments.

The effect of polygodial on cytoplasmic membrane integrity was examined by using the SYTOX Green uptake assay. The results are presented in Figure 6.

It is known that ethanol (70% *v*/*v*) causes cell membrane dehydration and therefore it is used as positive control. For ethanol-treated hyphae, fluorescent nuclei were observed, indicating alteration of the membrane (Figure 6A,B). On the other hand, methanol, used as negative control, has no effect on cell membrane and, therefore, the hyphae nuclei exhibited no fluorescence (Figure 6C,D). Conidia of *B. cinerea* treated with polygodial at 117 ppm after incubation for 1 h show fluorescent nuclei (Figure 6E,F). Therefore, polygodial induces membrane damage in the fungus.

**Figure 7:**
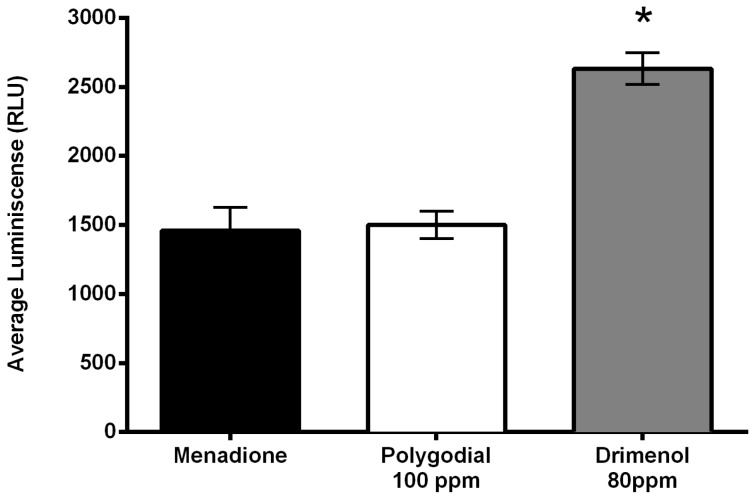
Effect of polygodial on production of reactive oxygen species by *Botrytis cinerea*. Measured luminescence was converted to concentration of H_2_O_2_ after background correction using a standard curve. Menadione (10 ppm final concentration) was used as positive control. (*) represents significant difference between mean of drimenol and means of the other two, at the 0.05 significance level. Data are from Robles-Kelly et al. [19].

In the same way, the effect of polygodial on reactive oxygen species (ROS) production was examined by incubating *B. cinerea* conidia in the presence of polygodial for 2 h at 21 °C. The production of ROS was evaluated using ROS-Glo™ H_2_O_2_ Assay as described by Robles-Kelly et al. [19]. In Figure 7 are shown the luminescence yields measured in presence of polygodial (100 ppm), drimenol (80 ppm) and menadione that is used as positive control. The results indicate that under these conditions polygodial induces a ROS production similar to menadione, but much lower than that exhibited by drimenol.

**Figure 8:**
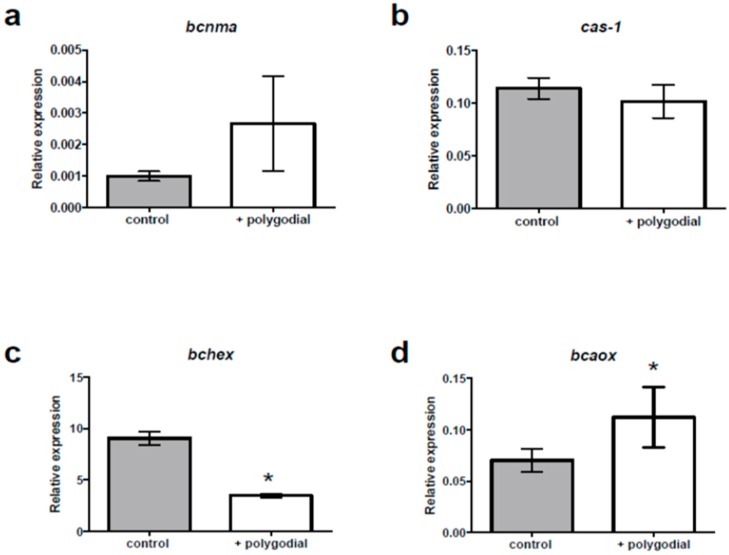
Effect of polygodial on gene expression of *Botrytis cinerea.* mRNA levels of *b*c*nma* (**a**), *cas-1* (**b**), *b*c*hex* (**c**), and *b*c*aox* (**d**) genes were measured in presence and the absence of polygodial after 1 h of incubation. All values represent the mean ± standard deviation of three independent experiments with at least three replicates each. (*) Represents experiments with a significant difference between control and polygodial treatments at the 0.05 significance level (Student’s *t*-test).

Finally, to obtain a better understanding on the mechanism by which polygodial acts against *B. cinerea*, changes in the expression levels of specific genes associated with cellular damage have also been studied. The results are shown in Figure 8.

The results show that genes *bcnma* and *cas-1*, which are associated with programmed death cell (PDC), exhibit no increase of transcript accumulation in presence of polygodial.

On the other hand, the expression of *bcaox*1, a gene related to oxidative stress in fungi, is increased in presence of polygodial, which suggest a mitochondrial dysfunction due to damage of ATP-forming complexes located in the inner membrane (Figure 8d). Finally, in Figure 8c it is clearly seen that polygodial induces a strong underexpression of *bchex*, which is a gene involved in hyphal growth damage [20].

## 3. Discussion

Herein, we discuss the results obtained in a study of antifungal activity of polygodial against *B. cinerea*. This secondary metabolite is a drimane sesquiterpene dialdehyde obtained from Canelo, which has previously shown interesting biological activities.

**Figure 9:**
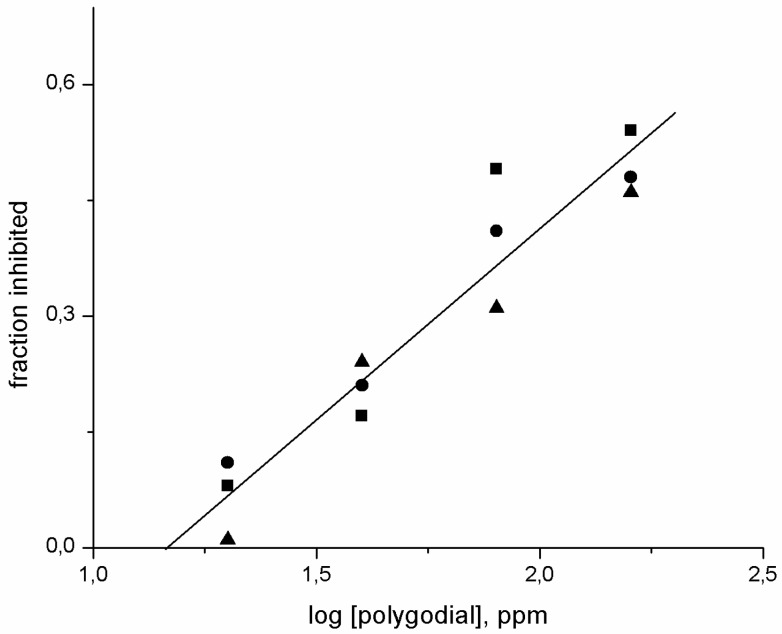
Fraction of inhibited mycelial growth as a function of polygodial concentration: (■) B05.10; (●) PN2; (▲) 7AC.

The antifungal activity of polygodial was evaluated by measuring growth inhibition of isolates with different degrees of sensitivity to commercial fungicides. In Figure 9 are shown the fraction of inhibited mycelial growth, determined for all isolates, as a function of polygodial concentration. It is seen that a good linear fit is obtained for the data. This result indicates that polygodial inhibits hyphal growth of common B05.10 and native isolates, which have developed resistance to fenhexamide and other commercial fungicides, with similar efficiency. This is an expected result and it might be interesting to determine how long it takes to these isolates to develop resistance to polygodial.

In addition, polygodial presents an EC_50_ of 117 ppm, which is higher than those reported for drimenol and its derivatives (80 ppm) [19] and for a commercial natural fungicide, BC-1000 (30 ppm). These results are quite interesting because polygodial is 15–30 times more active than drimenol on human yeast [18]. A comparison of chemical structures for these compounds indicates that the main difference rest on the electronic effect of the C-8 substituent on the double bond, i.e., the electronic density of this bond is increased by methyl, and decreased by aldehyde, substitution on C-8. Therefore, the inhibitory effect of these compounds on *B. cinerea* growth seems to be mainly determined by the double bond electronic density. 

On the other hand, germination results indicate that germination of *B. cinerea* is strongly reduced in the presence of polygodial, and that this effect is mainly produced at early stages of germination. Results shown in Figure 4 indicate that in the presence of polygodial (80 ppm) there is no germination of *B. cinerea*, whereas a 40% of germinated conidia is observed in the absence of this compound after 3 h of incubation. This inhibition effect on germination is much higher than that reported for other antifungal agents [19,21,22]. For example, after 6 h of incubation polygodial at 80 ppm decreases the germination to 5%, whereas under the same conditions drimenol decreases germination to 48% (see Figure 4). These results suggest that the effect of polygodial on hyphal growth, measured after 48 h, could be mainly a consequence of inhibition of *Botrytis* germination. In other words, it seems that polygodial acts mainly to delay the germination process, which ends by affecting the hyphal growth of *B. cinerea*. This result is completely different to what has been described for drimenol, which inhibits both the hyphal growth and conidia germination [19]. Consequently, it could be concluded that both compounds act against *B. cinerea* through different mechanisms.

To identify the mechanism of action of polygodial, a determination of membrane integrity, reactive oxygen species production, and gene expression studies of specific genes were performed. The results obtained in a study of membrane integrity indicate that polygodial disrupts the *B. cinerea* plasmatic membrane, enhancing entry of SYTOX Green into the nuclei. This agrees with previous studies of the polygodial effect on *Saccharomyces cerevisiae*, where it has been shown that plasma membrane disruption is the mechanism of polygodial-induced cell death [15]. Similar results have been reported for drimenol and 3β-hydroxykaurenoic acid, a diterpene that inhibits the growth of *B. cinerea* by disrupting the plasmatic membrane [19,21]. 

Induction of ROS production can be taken as a manifestation of hyphal damage. The results indicate that ROS production induced by polygodial is similar to that measured for menadione (positive control) but much lower than that reported for drimenol (Figure 7). These results confirm that the mechanism of action is different for both compounds. 

Regarding the gene expression studies, the results show that there is no overexpression of genes *bcnma* and *cas-1* (see Figure 8a,b). A negative transcriptional modulation of these genes suggests that apoptosis mechanism is not involved in polygodial mode of action. A similar result has been reported for drimenol [19]. On the other hand, the expression of *bcaox*1, a gene related to oxidative stress in fungi, is increased in the presence of polygodial suggesting a mitochondrial dysfunction due to damage of ATP-forming complexes located in the inner membrane (Figure 8d). However, the values of relative expression of this gene are much lower for polygodial than those reported for drimenol [19]. Finally, in the presence of polygodial the expression level of *bchex* is 4–5 times below the expression measured for the control, whereas in the presence of drimenol this gene is 20 times overexpressed. This gene encodes to the principal protein of the Woronin body whose main function is to seal septal pores in response to cellular damage, and in a previous work we have shown that growth of fungi mutation lacking this gene is greatly reduced [20]. Thus, these results confirm that polygodial is not affecting mycelial growth and, therefore, its effect may be completely attributed to inhibition of germination. Agreeing with this observation, the major effect of polygodial appears at the early stages of *B. cinerea* development (Figure 4).

Currently, a robust gene expression study using RNA-seq technology is being performed to elucidate the specific mechanism by which polygodial affects *B. cinerea* and to clarify the real participation in germination process.

## 4. Materials and Methods

### 4.1. Chemistry

#### Extraction and Purification of Polygodial from *D. Winteri*

Polygodial was obtained from *Drymis winteri* barks following a procedure described by Cechinel et al., (1998) [11]. Briefly, dry canelo bark (*Drymis winteri*) collected in the area of Valdivia, Los Rios, Chile, was macerated in ethyl acetate for three days; the extract was concentrated under reduced pressure and then purified by column chromatography using mixtures of hexane/ethyl acetate of increasing polarity. Fractions composition was monitored by thin layer chromatography (TLC) using an authentic polygodial sample. Those polygodial-enriched fractions were collected and passed through silica gel column to obtain pure polygodial. Chemical structure of this compound was confirmed by spectroscopic methods (IR, HNMR H and C) and compared with a standard sample.

### 4.2. Biological

#### 4.2.1. Fungal Isolate and Culture Conditions

B05.10, PN2, and 7AC isolates of *B. cinerea* used in this study were maintained and grown under conditions previously described [19]. The main characteristics of isolates used in this study are shown in Table 1.

#### 4.2.2. Effect of Polygodial on Mycelial Growth of *B. cinerea* in Solid Media

Fungitoxicity of polygodial and of commercial fungicide BC-1000 was assessed using the radial growth test on malt-yeast extract agar. Polygodial was dissolved in dichloromethane to obtain different final concentrations (20, 40, 80, 160 ppm). Polygodial was completely soluble at all concentrations tested. The procedure was similar to that described by Robles-Kelly et al. [19]. Significant differences were evaluated with a two-way analysis of variance (Tukey’s test; *p* < 0.05). EC_50_ values of both fungicides for mycelial growth of *B. cinerea* isolates were analysed through the PROBIT Test using the MINITAB V.16 program (Minitab Inc., State College, PA, USA).

#### 4.2.3. Effect of Polygodial on Germination of *B*. *cinerea* Conidia

Conidial germination assays were carried out following a previously-described procedure [16]. Briefly, polygodial dissolved in dichloromethane was added to microscope slides coated with soft agar medium at final concentrations of 20, 40, 80 and 160 ppm. The percentage of germination was estimated by counting the number of germinated conidia, and judging as germinated those conidia where the germ tube length was equal to or greater than conidial diameter. Each experiment was performed in triplicate.

### 4.3. Mode of Action of Polygodial on B. cinerea

#### 4.3.1. Determination of Membrane Integrity of *B. cinerea*

The integrity of *B. cinerea* cytoplasmic membrane was determined, in the presence and absence of polygodial, by using SYTOX Green uptake assay [19]. Briefly, *B. cinerea* conidia germinated, after incubation for 16 h at 23 °C, were washed and incubated in a minimal medium supplemented with 117 ppm of polygodial for 3 h. After that, conidia were washed and incubated in presence of SYTOX Green reactive. Finally, SYTOX Green fluorescence was analyzed by microscopy. Ethanol (70%) and methanol (100%) were used as positive and negative controls, respectively. In addition, a solvent control was also utilized.

#### 4.3.2. Production of Reactive Oxygen Species

Polygodial-induced production of reactive oxygen species (ROS) was evaluated using ROS-GloTM H_2_O_2_ assay kit (Promega, Madison, WI, USA) [19]. Spores (79 µL) were plated in a 96-well plate at a concentration of 1 × 10^5^ spores/mL/well. Later, each well was incubated in presence of 1 µL polygodial (117 ppm), 20 µL of buffer substrate H_2_O_2_, and cultured for 2 h at 21 °C. After this period of incubation, 100 µL of ROS-GloTM reagent was added to each well and incubated for 20 min at room temperature. ROS production was measured using a luminometer (Tecan infinite m200pro). Menadione (10 ppm) was used as positive control, following the manufacturer's instructions. The results were subjected to Student’s *t*-test to verify the existence of differences at the 0.05 significance level.

#### 4.3.3. Real-Time PCR and Gene Expression

For gene expression analysis, conidia were inoculated in sterile Erlenmeyer flasks with malt-yeast medium and cultured without stirring for 6 h at 21 ± 1 °C in the presence of 117 ppm of polygodial. Then, conidia was extracted and grounded with liquid nitrogen. Total RNA extraction and cDNA synthesis was performed following a described procedure [23]. At least three independent cDNA preparations obtained from independent biological replicates were used for analysis. Each target gene was measured in triplicate and the mean Ct value was calculated. Expression of each gene was determined by the method of Livak and Schmittgen [24]. Expression levels were normalized against a reference gene, namely ubiquitin-conjugating enzyme E2 (*ubce*). Primers and amplification efficiencies (E) are listed in Table S1 (Supplementary Material). Significant differences between control and polygodial treatments were evaluated with a one-way analysis of variance (Genstat 5, Release 4.1). Means values were subjected to Student *t*-test to verify the existence of differences at the 0.05 significance level.

## 5. Conclusions

The antifungal activity of polygodial on hyphal growth and germination of *B. cinerea* has been evaluated. Our results suggest that polygodial affects mycelial growth and powerfully inhibits the germination of *B. cinerea*. The mechanism of action of this molecule involves destabilization of membrane integrity, ROS production, and overexpression of genes associated with oxidative stress on *B. cinerea*. Comparison of these results with those obtained for drimenol allows one to conclude that the mechanisms of both compounds are completely different, i.e., polygodial acts at early stages of germination, whereas drimenol inhibits the mycelial growth.

## Figures and Tables

**Table 1:** Characteristics of *Botrytis cinerea* isolates used in this study.

Isolate	Host	Resistance
B05.10	Grape	none
PN2	Cherry	Fenhexamid, Iprodione, Pyrimethanil, and Boscalid
7AC	Pear	Fenhexamid

## Supplementary Materials

Supplementary materials can be found at www.mdpi.com/1422-0067/18/11/2251/s1.
