# Supplementary material for: Antifungal Effect of Polygodial on Botrytis cinerea, a Fungal Pathogen Affecting Table Grapes
Source: Int J Mol Sci. 2017 Oct 27;18(11):2251. doi: 10.3390/ijms18112251 (PMC5713221; doi:10.3390/ijms18112251)
Supplement: Supplementary file 1 [file ijms-18-02251-s001.pdf]

Supplementary Material: Antifungal Effect of Polygodial on *Botrytis cinerea*, a Fungal Pathogen Affecting Table Grapes

Héctor Carrasco, Christian Robles-Kelly, Julia Rubio, Andrés F. Olea, Rolando Martínez and Evelyn Silva-Moreno

Table 1. Primers and their amplification efficiency

Primer		Sequence (5´-3´)	Tm, °C	qPCR Efficiency
bchex	Forward	tctacttcaacgaggcttc	57	99
	Reverse	caccagattgaccgaaaac		
bcnma	Forward	atgatcggcaccaacggcgt	57	98
	Reverse	caaaggtcgatcgagtggcaa		
cas-1	Forward	atggaggctatggagcacca	57	99
	Reverse	gcaacaccgctcatatcacct		
bcaox	Forward	tgcatgtactgcacctacaccatcg	57	100
	Reverse	gacgcgatcggaaaaatctcg		
ubce	Forward	catcaactccaacggaagca	57	100
	Reverse	tcggtcggtcttgtaaacgt		
